# Daily Consumption of Virgin Coconut Oil Increases High-Density Lipoprotein Cholesterol Levels in Healthy Volunteers: A Randomized Crossover Trial

**DOI:** 10.1155/2017/7251562

**Published:** 2017-12-14

**Authors:** Surarong Chinwong, Dujrudee Chinwong, Ampica Mangklabruks

**Affiliations:** ^1^Department of Pharmaceutical Care, Faculty of Pharmacy, Chiang Mai University, Chiang Mai 50200, Thailand; ^2^Department of Internal Medicine, Faculty of Medicine, Chiang Mai University, Chiang Mai 50200, Thailand

## Abstract

This open-label, randomized, controlled, crossover trial assessed the effect of daily virgin coconut oil (VCO) consumption on plasma lipoproteins levels and adverse events. The study population was 35 healthy Thai volunteers, aged 18–25. At entry, participants were randomly allocated to receive either (i) 15 mL VCO or (ii) 15 mL 2% carboxymethylcellulose (CMC) solution (as control), twice daily, for 8 weeks. After 8 weeks, participants had an 8-week washout period and then crossed over to take the alternative regimen for 8 weeks. Plasma lipoproteins levels were measured in participants at baseline, week-8, week-16, and week-24 follow-up visits.* Results*. Of 32 volunteers with complete follow-up (16 males and 16 females), daily VCO intake significantly increased high-density lipoprotein cholesterol by 5.72 mg/dL (*p* = 0.001) compared to the control regimen. However, there was no difference in the change in total cholesterol, low-density lipoprotein cholesterol, and triglyceride levels between the two regimens. Mild diarrhea was reported by some volunteers when taking VCO, but no serious adverse events were reported.* Conclusion*. Daily consumption of 30 mL VCO in young healthy adults significantly increased high-density lipoprotein cholesterol. No major safety issues of taking VCO daily for 8 weeks were reported.

## 1. Introduction

Consumption of saturated fatty acids (SFAs) in rich diets has been attributed to increased risk of cardiovascular disease (CVD) [1]. In populations consuming a Western diet, the replacement of 1% of energy from SFAs with polyunsaturated fatty acids (PUFAs) can lower LDL-cholesterol and subsequently reduce the incidence of CVD by 2-3% [2]. Indeed, several guidelines for the prevention of cardiovascular disease recommend decreasing the intake of SFAs to reduce the risk of developing CVD [1, 3–6]. Coconut oil contains a high amount of SFAs and for this reason its consumption should be reduced to a maximum of 10% of total energy intake [1]. Coconut products (coconut meat, milk, and oil) have been consumed as part of traditional food in Thailand, but its consumption has declined over the last 20 years as people have been informed they should avoid consuming foods high in SFAs. Nevertheless, the prevalence of CVD and risk factors for CVD in the Thai population have increased over this period of time [7, 8]. A review of population studies by Kaunitz and Dayrit in 1992 found that consumption of dietary coconut oil did not lead to higher serum cholesterol levels nor to a higher rate of mortality or morbidity related to CVD [9]. Coconut oil is now receiving attention as a functional food oil [10] and its consumption has dramatically risen in recent years. Coconut oil, especially virgin coconut oil (VCO), has been claimed to have beneficial effects on health [11, 12] and there are multiple product literature sources, magazine articles, web sites, and books promoting its use [13]. Unfortunately, the claims made for VCO are not supported by robust scientific evidence [14] and as yet there has been no published study demonstrating the benefit of coconut oil to cardiovascular disease outcome [15]. A few published studies have investigated the effect of coconut oil on lipid profiles in adults [16–26]. Eight studies have reported that coconut oil consumption significantly increased total cholesterol levels compared to baseline levels or a comparator, that is, safflower oil, soybean oil, palm oil, corn oil, and olive oil [16–23], while seven studies found that coconut oil significantly increased low-density lipoprotein cholesterol (LDL-C) levels [16–18, 20–23]. An observational study and a meta-analysis of individual data have shown that high-density lipoprotein cholesterol (HDL-C) levels are inversely associated with risk of CVD and cardiovascular mortality [27–29], and in six of these coconut oil studies HDL-C levels significantly increased [17, 18, 21–23, 26]. One observational study showed that dietary coconut oil intake was positively associated with total cholesterol and HDL-C levels among premenopausal women [24].

To date, there has been no scientific study assessing the benefit of coconut oil in the Thai population. This study aimed to investigate the effect of daily VCO consumption on plasma lipoproteins levels and document any adverse effects.

## 2. Materials and Methods 

### 2.1. Setting and Study Population

This study was conducted at the Faculty of Pharmacy, Chiang Mai University, Thailand. The study protocol was approved by the Ethical Review Committee, Faculty of Pharmacy, Chiang Mai University, Thailand, and was conducted in accordance with the Declaration of Helsinki and Good Clinical Practice.

Inclusion criteria for volunteers were (1) healthy male or female, (2) age from 18 to 25, (3) no history of allergy to coconut oil and/or carboxymethylcellulose (CMC), (4) not taking any medications, (5) having all related biomedical parameters in the normal range, (6) ability to provide informed consent and willingness to take VCO and 2% carboxymethylcellulose solution (2% CMC solution) as per protocol, and (7) agreement on follow-up for the duration of the study. Exclusion criteria included (1) volunteers unwillingness to have their biomedical parameters assessed and (2) being pregnant. A total of 35 Thai healthy volunteers were recruited. The participants were free to decide whether to volunteer for the study and written informed consent was obtained from all volunteers before starting the study.

### 2.2. Study Design

This was an open-label, randomized, controlled, crossover study. The crossover design study had two periods, each lasting for eight weeks, separated by an eight-week washout period. The principal investigator was responsible for randomizing each participant by a simple random method.

**Figure 1:**
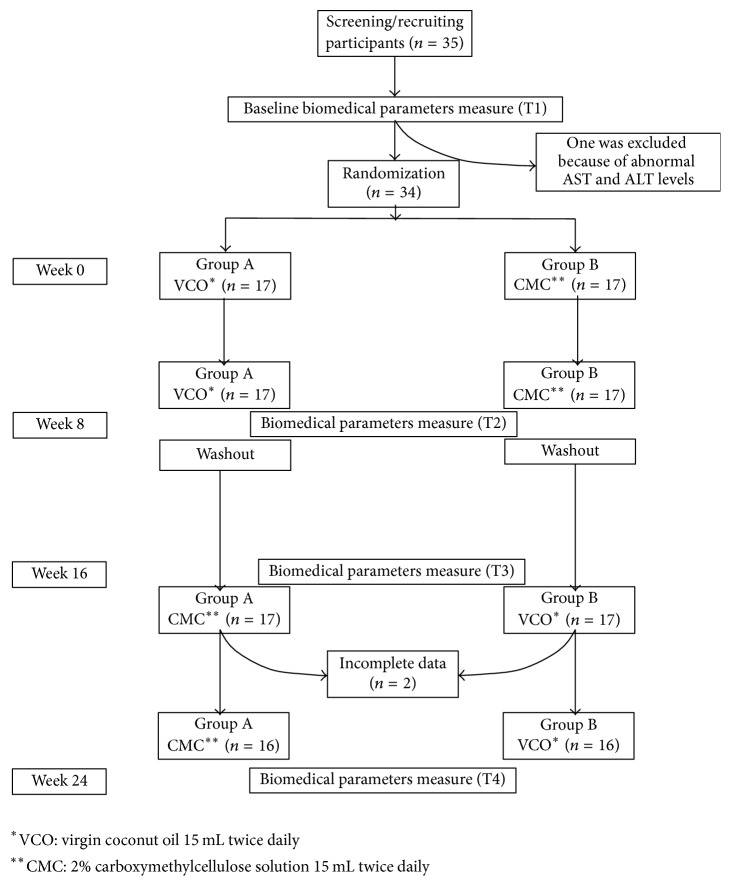
Flow diagram of the open-label, randomized, controlled, crossover study comparing the effects of consuming virgin coconut oil 15 mL twice daily and 2% carboxymethylcellulose solution (as a control) for 8 weeks among healthy volunteers.

At baseline (week 0), participants were randomized to take either 15 mL VCO or 2% CMC solution (as control), twice daily, for 8 weeks. Relevant biomedical parameters and lipid profiles (total cholesterol, LDL-C, HDL-C, and triglyceride) were determined for each participant at the baseline visit and then again at week 8. After the 8-week visit participants stopped taking their assigned regimen and had a “washout” period for 8 weeks. At week 16, participants crossed over to take the alternative regimen for 8 weeks. Biomedical parameters were measured prior to restarting the new regimen and at the week-24 study visit (Figure 1). The advantage of a crossover design is that each participant is acting as their own control. For this reason it was important that the participants continued with the same diet while taking each of the study regimens to ensure that any difference in lipid profiles observed was likely due to the study regimen rather than a change in individual diet.

### 2.3. Products Used in the Study

VCO was produced by Siam Paradise Health Products Co. Ltd. The oil contained lauric acid (C12:0) 50.4%, myristic acid (C14:0) 17.5%, caprylic acid (C8:0) 9.0%, palmitic acid (C16:0) 7.9%, capric acid (C10:0) 7.0%, oleic acid (C18:1n9c) 5.9%, stearic acid (C18:0) 1.6%, and linoleic acid (C18:2n6) 0.9%. The control regimen was 2% CMC solution, produced by the Faculty of Pharmacy, Chiang Mai University. A 2% CMC solution was chosen as the control regimen instead of other types of oil in order to avoid any effects of other oils on the lipid profiles.

### 2.4. Compliance of Participants

Participants were requested to maintain their usual habitual pattern of diet and physical activity during the study period. All participants started taking VCO and 2% CMC solution at the same date as per protocol. To assess their compliance, all participants were asked to record their daily consumption, for example, food, food supplement, medicines, activities, and any presented adverse effects throughout the study period in a study diary. In addition, participants were interviewed regarding their diet, exercise, and compliance with intake of the VCO and control regimens.

### 2.5. Efficacy Outcomes Measured

The primary efficacy outcome was the change in plasma lipoproteins levels from baseline to the end of the treatment with VCO versus 2% CMC solution (control). The plasma lipoproteins assessed were total cholesterol, triglyceride, HDL-C, and LDL-C.

### 2.6. Safety Outcomes Measured

To ensure the safety of participants, measurement of related biomedical parameters—blood urea nitrogen (BUN), serum creatinine (SCr), aspartate aminotransferase (AST), alanine aminotransferase (ALT), and alkaline phosphatase—was performed at baseline (week 0), week 8, week 16, and week 24. Participants with results out of the normal range were excluded from the study before randomization.

### 2.7. Laboratory Investigation

A standard protocol for fasting was provided to each participant. All participants were asked to fast overnight for at least 12 hours before blood samples were drawn. Fasting blood samples were taken by venipuncture at baseline (week 0), week 8, week 16, and week 24. Blood samples were generally taken by the same person and at the same location. All blood tests were performed at the Clinical Service Center in the Faculty of Associated Medical Sciences, Chiang Mai University. Biomedical parameters—BUN, SCr, AST, ALT, and alkaline phosphatase—and a lipid profile—total cholesterol, triglyceride, HDL-C, and LDL-C (calculated by the Friedewald formula)—were measured at baseline (week 0), week 8, week 16, and week 24. Body weight, body mass index, and blood pressure were measured at the same time as blood sample collection.

### 2.8. Sample Size Calculation

The sample size calculation was based on the hypothesis that taking VCO 30 mL/day would increase HDL-C level by 10%. A mean baseline HDL-C level (±SD) of 45.5 ± 7.1 mg/dL was taken based on the report by Assunção et al. [25]. Assuming a standard deviation of 7.1, a sample size of 40 participants was needed to conclude a difference of HDL-C of 4.6 mg/dL (calculated for a two-tailed test with a type I error of 0.05 and a power of 0.80); for a crossover study design, see http://hedwig.mgh.harvard.edu/sample_size/js/js_crossover_quant.html.

### 2.9. Statistical Analysis

Stata software version 12 (StataCorp LP, College Station, TX, USA) was used for all statistical analyses. Descriptive statistics were used for the characteristics of participants; continuous variables were reported as means with standard deviations and categorical variables as frequencies and percentages. A carry-over effect was tested by comparing the difference between the participants' response to VCO and 2% CMC solution (control) at week 8 with the difference between the participants' response to VCO and control at week 24 using a two-sample *t*-test. A treatment effect of VCO was tested using a paired *t*-test to compare the mean difference in change in plasma lipoproteins levels between VCO and 2% CMC solution (control). A *p* < 0.05 was deemed statistically significant for all analyses.

## 3. Results

### 3.1. Characteristics of Participants

Thirty-five healthy volunteers were recruited. One volunteer was excluded before randomization because of AST and ALT levels above the normal range at baseline (week 0, Figure 1). Two volunteers were unable to be followed up at the last study visit (week 24) and were excluded from the analysis. Of 32 participants (16 males and 16 females) who completed the study, the mean age ± SD was 21.0 ± 0.74 years (range: from 18 to 25 years) and BMI was 20.8 ± 3.43 kg/m^2^. The characteristics of participants are shown in Table 1.

### 3.2. The Effects of VCO on Plasma Lipoproteins Levels

No carry-over effect was observed. Among these healthy participants taking 15 mL of VCO twice daily for 8 weeks, the mean HDL-C level significantly increased from 60.3 ± 9.2 mg/dL to 64.2 ± 9.9 mg/dL (*p* = 0.001), while the HDL-C level did not significantly change after taking 2% CMC solution. However, taking 2% CMC solution significantly reduced total cholesterol level from 191.1 ± 32.1 mg/dL to 183.7 ± 33.7 mg/dL (*p* = 0.021) and LDL-C level from 116.4 ± 30.2 mg/dL to 110.2 ± 31.7 mg/dL (*p* = 0.036), while the levels of total cholesterol and LDL-C did not significantly change after taking VCO (Table 2).

VCO twice daily was associated with a significant increase in HDL-C level by 5.72 ± 9.01 mg/dL (*p* = 0.001, 95% CI: 2.44–9.00). The changes in total cholesterol, LDL-C, and triglyceride levels did not significantly differ between the two regimens (Table 3).

### 3.3. The Adverse Effects of VCO

In general, no significant changes were observed from baseline in blood pressure, body weight, renal function (according to SCr and BUN levels), and hepatic functions (according to AST, ALT, and alkaline phosphatase levels) among the participants while taking either VCO or 2% CMC solution (Table 2). Although the change in ALT levels was statistically significantly higher when taking coconut oil, compared to that when taking 2% CMC solution (Table 3); this change was not thought to be clinically significant.

Some participants reported mild diarrhea or loose stool, especially during the first week of taking coconut oil and/or taking it on an empty stomach (Table 4). However, these symptoms were resolved in many participants after the second week.

## 4. Discussion

### 4.1. The Effects of VCO on Plasma Lipoproteins Levels

This open-label, randomized, controlled, crossover trial among 32 healthy participants assessed the effects of daily VCO consumption on plasma lipoproteins levels compared to control (2% CMC solution). We found that taking 15 mL of VCO twice daily for 8 weeks was associated with a significant increase in HDL-C level compared with taking 2% CMC solution. Nevertheless, we did not find any significant differences in total cholesterol, LDL-C, and triglyceride levels between VCO and the control.

Our findings are in line with the findings that VCO consumption is potentially beneficial for increasing HDL-C levels [17, 18, 21–23, 26]. Our lipid parameter results are similar to a recent study by Cardoso et al. who evaluated the effect of VCO on lipid profiles and anthropometric parameters among patients with coronary artery disease (CAD). Cardoso et al. found that HDL-C levels significantly increased (*p* < 0.01) in those consuming VCO 15 mL for 3 months, while no significant change occurred in total cholesterol or LDL-C levels [26]. Our results are similar to a randomized crossover (3 × 3 Latin-square) study by Voon et al. conducted in Malaysia with 45 healthy participants who consumed diets with 30% of energy from fat and two-thirds of fat from coconut oil, palm oil, or extra virgin olive oil [23]. Compared with consuming extra virgin olive oil, consuming coconut oil significantly increased the HDL-C level by 3.48 mg/dL (*p* < 0.05) [23].

A possible explanation for the increase in HDL-C observed in our study may be due to the high proportion of lauric acid and myristic acid in the VCO product used. In general, diets high in saturated fat raise HDL-C level [30, 31], while replacing saturated fat with polyunsaturated or monounsaturated fat lowers HDL-C [32]. A meta-analysis of 27 trials evaluated effects of three classes of fatty acids, that is, saturated, monounsaturated, and polyunsaturated fatty acids, on serum lipid and lipoprotein levels. Between these three classes of fatty acids, saturated fatty acid had the greatest raising effect on HDL-C, total cholesterol, and LDL-C. In contrast, monounsaturated and polyunsaturated fatty acids lowered total cholesterol and LDL-C levels [33]. Moreover, among saturated fatty acids, a study compared the effects of diets high in caprylic acid (C8:0) plus capric acid (C10:0) and diets high in lauric acid (C12:O) on lipid metabolism. It was shown that the total HDL-C level was significantly increased (*p* = 0.002) in diets rich in lauric acid, whereas the level of HDL-C did not change in diet rich in caprylic acid plus capric acid [34]. Most of the saturated fats in coconut oil are medium chain triglyceride (MCT), containing 6 to 12 carbon fatty acids [12, 35]. The composition of saturated fatty acids in VCO used in our study was caprylic acid (C8:0) 9.0%, capric acid (C10:0) 7.0%, lauric acid (C12:0) 50.4%, myristic acid (C14:0) 17.5%, palmitic acid (C16:0) 7.9%, and stearic acid (C18:0) 1.6%. Furthermore, Vaysse-Boué et al. demonstrated that diets higher in myristic acid significantly increased lecithin-cholesterol acyltransferase (LCAT) activity more than diets lower in myristic acid. LCAT has a significant role in regulating HDL-C level [36]; thus a high proportion of lauric acid and myristic acid in VCO may explain the increase in HDL-C observed in our study.

Our results differed from a study by Voon et al. that found a significant increase in total cholesterol and LDL-C levels (11.58 mg/dL (*p* < 0.05) and 9.27 mg/dL (*p* < 0.05), respectively), when two-thirds of the total dietary fat in the participants' diet was replaced by coconut oil [23]. An important difference between our study and that of Voon et al. was that the composition of fat in the participants' diet was changed, while in our study participants received VCO on top of their regular dietary food. Another six studies have also demonstrated that coconut oil significantly increases low-density lipoprotein cholesterol (LDL-C) level when compared with oils containing unsaturated fat [16–18, 20–22]. A study by Tholstrup et al. comparing the effect of taking 70 g of MCTs (66% C8:0 and 34% C10:0) and high-oleic sunflower oil (HOSO) (89.4% C18:1n9c) found that intake of MCTs resulted in a 12% higher (11.6 mg/dL) LDL-C level (*p* = 0.0001) [37]. In our study, the composition of C8:0 and C10:0 accounted for only 16% of fatty acids contained in the VCO. Moreover, we compared the LDL-C level between those taking VCO and control (2% CMC), while the majority of other studies compared VCO with an unsaturated fat as the control. This difference may explain why the intake of VCO in our study did not result in a significant increase in LDL-C level. However, a study in healthy men demonstrated that an increase in intake of total saturated fatty acid (especially myristic acid and palmitic) elevated the LDL-C level, mainly due to an increase in plasma levels of larger LDL particles while the level of smaller LDL particles was decreased [38]. Clearly, evaluating the effect of nutrition and diet on LDL-C level alone may not be sufficient but unfortunately in our study we did not assess the levels of LDL subclasses.

A recent randomized, double-blind, study conducted in Brazil involving 40 healthy women with abdominal obesity who received daily supplements with either 30 mL of soybean oil or coconut oil found that, after a 12-week intervention, there was no significant change in lipid parameters in those consuming coconut oil. The levels of total cholesterol and LDL-C significantly increased (*p* < 0.01) in those taking soybean oil whereas the level of HDL-C significantly reduced (*p* = 0.04) [25].

### 4.2. The Adverse Effects of VCO

Interestingly, our study did not reveal any change in weight or BMI from baseline after taking VCO for 8 weeks. It should be noted that the participants in our study were asked to take 30 mL of VCO daily in addition to their regular dietary food. Considering the total amount of VCO, each participant in our study consumed approximately 1,680 mL of VCO throughout 8-week period. This amount of VCO can be converted to energy of about 14,000 kcal [35] and according to “3,500 kcal per pound weight loss rule,” this amount of energy would translate into a weight gain of around 4 pounds or 1.8 kg [39]. It is possible that the duration of the study might have been too short to observe any changes in weight. Moreover, the baseline weights of participants were relatively low. Another possible reason for the absence of weight gain in our study may be explained by the high composition of MCT in VCO. MCT is absorbed directly into the portal circulation and transported to the liver for rapid oxidation. Human studies have found that short-term consumption of MCT increases energy expenditure. Studies have also shown that replacing dietary long chain triglyceride (LCT) with MCT increased total energy expenditure from 24 to 160 kcal/d in males [40] and from 31 to 45 kcal/d in females [41]. A study evaluating postprandial thermogenesis (PTT) after the ingestion of a mixed meal containing either LCT 38 g (LCT meal) or MCT 30 g plus LCT 8 g (MCT meal) revealed that PTT (0 to 6 hr) after MCT meal was 48.5% and 65.1%, respectively, significantly higher than PTT after LCT meal in lean and obese subjects [42]. Moreover, consuming MCT may enhance satiety and decrease food intake. A study evaluating the effect of breakfasts differing in the nature of the fat, that is, monounsaturated LCT, saturated LCT, or MCT, on food intake found that energy intake at lunch was lower in adults after the MCT-containing breakfast than those after all other breakfasts [43]. This could also be a potential explanation for the stability of participants' weight in our study.

Regarding participant safety, we found that eating 15 mL of VCO twice daily for 8 weeks was not associated with changes in AST or ALT levels or SCr and BUN. A small increase in ALT level was found among those taking VCO compared to those taking 2% CMC solution. This small increase in ALT level was still within the normal range (10–40 U/L) and deemed not clinically significant. No participants had ALT levels higher than the normal range.

Some small side effects from taking VCO were observed. Of 32 participants, the side effects of taking VCO included diarrhea or loose stool (71.9%), mild stomach ache (19%), and vomiting (16%). These side effects were normally reported in the first week after starting VCO; however, the symptoms normally resolved by the second week. None of the side effects had an impact on the daily life activities of the participants.

### 4.3. Strengths and Limitations

Our study has several strengths. Firstly, the crossover design employed allowed individuals to act as their own control and thus removed intersubject variability. We also allowed 8 weeks for the washout period, allowing sufficient time to minimize the impact of any carry-over effect. Secondly, VCO was provided as a supplement to the daily food intake of each participant. Thirdly, all participants were asked to maintain their usual habits and food intake during the coconut oil and control periods.

However, some limitations should also be acknowledged. This was an open-label study because of the difference in the appearance of VCO and 2% CMC. This study was only conducted with healthy volunteers aged 18 to 25; therefore, the generalizability of the findings should be used with caution among people outside this age range or patients, particularly those with comorbidities. Thus, further research should be conducted in the real-world practice among patients with low HDL-C levels, especially those at high risk of cardiovascular events. For example, a future study could investigate the effect of coconut oil among patients with low HDL-C levels with or without diabetes mellitus, who are at high risk of cardiovascular disease.

## 5. Conclusion

We found an increase in HDL-C levels among young healthy volunteers taking dietary supplements with 15 mL of VCO twice daily, as compared with taking control (2% CMC solution), and found no significant harmful side effects. The effect of VCO should be potentially beneficial for cardiovascular risk reduction but further studies are needed among patients with low HDL-C that need to increase their HDL-C levels.

## Figures and Tables

**Table 1:** Characteristics of participants who completed the study (*n* = 32).

Characteristics	Participants (mean ± SD)
Male/female (number)	16/16
Age (year)	21.0 ± 0.74
Weight (kg)	59.0 ± 11.87
BMI (kg/m^2^)	20.8 ± 3.43
SBP (mmHg)	115.1 ± 8.75
DBP (mmHg)	71.2 ± 7.37
BUN (mg/dL)	12.1 ± 3.12
SCr (mg/dL)	0.75 ± 0.18
Total cholesterol (mg/dL)	190.8 ± 32.29
Triglyceride (mg/dL)	68.5 ± 23.10
HDL-cholesterol (mg/dL)	60.6 ± 9.00
LDL-cholesterol (mg/dL)	116.5 ± 30.07
AST (U/L)	20.0 ± 9.21
ALT (U/L)	15.1 ± 6.20
AP (U/L)	70.3 ± 17.35

*Note*. To convert total, LDL-cholesterol and HDL-cholesterol levels from mg/dL to mmol/L, multiply by 0.02586; to convert triglyceride level from mg/dL to mmol/L, multiply by 0.01129. *Abbreviations*. BMI: body mass index; SBP: systolic blood pressure; DBP: diastolic blood pressure; BUN: blood urea nitrogen; SCr: serum creatinine; HDL: high density lipoprotein; LDL: low density lipoprotein; AST: aspartate aminotransferase; ALT: alanine aminotransferase; AP: alkaline phosphatase.

**Table 2:** Biomedical parameters results of 32 participants before and after consuming 15 mL of virgin coconut oil or 2% carboxymethylcellulose (CMC) solution twice daily for 8 weeks.

	Biomedical parameter (mean)					
	Virgin coconut oil			2% CMC solution		
	Before	After	*p* value^*∗*^	Before	After	*p* value^*∗*^
Total cholesterol (mg/dL)	190.4	187.7	0.389	191.1	183.7	0.021
Triglyceride (mg/dL)	67.8	64.7	0.477	69.3	72.3	0.493
HDL – cholesterol (mg/dL)	60.3	64.2	0.001	60.8	59.0	0.124
LDL – cholesterol (mg/dL)	116.6	110.5	0.061	116.4	110.2	0.036
Weight (kg)	58.9	59.2	0.365	59.1	58.7	0.430
BMI (kg/m^2^)	20.8	20.9	0.495	20.9	20.7	0.403
SBP (mmHg)	114.3	114.8	0.762	115.8	117.6	0.187
DBP (mmHg)	71.2	70.4	0.636	71.3	69.5	0.430
BUN (mg/dL)	12.3	12.1	0.835	12.0	11.2	0.066
SCr (mg/dL)	0.80	0.81	0.231	0.79	0.82	0.057
AST (U/L)	19.1	19.5	0.699	20.8	18.8	0.247
ALT (U/L)	15.0	17.2	0.105	15.2	14.4	0.517
AP (U/L)	71.7	71.2	0.683	68.9	69.4	0.807

*Note*. ^*∗*^Paired  *t*-test. To convert total, LDL-cholesterol, and HDL-cholesterol levels from mg/dL to mmol/L, multiply by 0.02586; to convert triglyceride level from mg/dL to mmol/L, multiply by 0.01129. *Abbreviations*. BMI: body mass index; SBP: systolic blood pressure; DBP: diastolic blood pressure; BUN: blood urea nitrogen; SCr: serum creatinine; HDL: high density lipoprotein; LDL: low density lipoprotein; AST: aspartate aminotransferase; ALT: alanine aminotransferase; AP: alkaline phosphatase.

**Table 3:** Differences in biomedical parameters before and after consuming 15 mL of virgin coconut oil or 2% carboxymethylcellulose (CMC) solution twice daily for 8 weeks (*n* = 32).

	Biomedical parameters (mean ± SD)			*p* value^*∗*^
	Change		Difference in change	
	Virgin coconut oil	2% CMC solution		
Total cholesterol (mg/dL)	−2.78 ± 18.00	−7.41 ± 17.26	4.63 ± 24.91	0.302
Triglyceride (mg/dL)	−3.06 ± 24.07	3.06 ± 25.00	−6.13 ± 32.70	0.298
HDL-cholesterol (mg/dL)	3.91 ± 6.34	−1.81 ± 6.49	5.72 ± 9.01	*0.001*
LDL-cholesterol (mg/dL)	−6.08 ± 17.65	−6.21 ± 16.04	0.13 ± 21.16	0.972
Weight (kg)	0.31 ± 1.92	−0.38 ± 2.65	0.72 ± 4.35	0.387
BMI (kg/m^2^)	0.08 ± 0.64	−0.15 ± 0.98	0.23 ± 1.15	0.278
SBP (mmHg)	0.50 ± 9.28	1.81 ± 7.61	−1.31 ± 10.52	0.485
DBP (mmHg)	−0.78 ± 9.24	−1.78 ± 12.60	1.00 ± 16.91	0.740
BUN (mg/dL)	−0.13 ± 3.36	−0.84 ± 2.50	0.72 ± 4.35	0.357
SCr (mg/dL)	0.02 ± 0.07	0.03 ± 0.09	−0.02 ± 0.10	0.393
AST (U/L)	0.38 ± 5.44	−2.00 ± 9.60	2.38 ± 10.80	0.223
ALT (U/L)	2.16 ± 7.30	−0.81 ± 7.02	2.97 ± 7.86	*0.041*
AP (U/L)	−0.47 ± 6.43	0.44 ± 10.04	−0.91 ± 7.86	0.705

*Note*. ^*∗*^Paired  *t*-test. To convert total, LDL-cholesterol, and HDL-cholesterol levels from mg/dL to mmol/L, multiply by 0.02586; to convert triglyceride level from mg/dL to mmol/L, multiply by 0.01129. *Abbreviations*. HDL: high density lipoprotein; LDL: low density lipoprotein; BMI: body mass index; SBP: systolic blood pressure; DBP: diastolic blood pressure; BUN: blood urea nitrogen; SCr: serum creatinine; AST: aspartate aminotransferase; ALT: alanine aminotransferase; AP: alkaline phosphatase.

**Table 4:** Self-reported adverse events from consuming 15 mL of virgin coconut oil or 2% carboxymethylcellulose (CMC) solution twice daily for 8 weeks (*n* = 32).

Events	Number of participants with events (%)	
	Virgin coconut oil	2% CMC solution
Diarrhea/loose stool	23 (71.9)	1 (3.1)
Stomach ache	6 (18.8)	1 (3.1)
Nausea/vomit	5 (15.6)	0 (0.0)
